# Hippocampal Disinhibition Reduces Contextual and Elemental Fear Conditioning While Sparing the Acquisition of Latent Inhibition

**DOI:** 10.1523/ENEURO.0270-21.2021

**Published:** 2022-01-19

**Authors:** Stuart A. Williams, Miriam Gwilt, Rebecca Hock, Charlotte Taylor, Joanna Loayza, Carl W. Stevenson, Helen J. Cassaday, Tobias Bast

**Affiliations:** 1 School of Psychology, University of Nottingham, Nottingham NG7 2RD, United Kingdom; 2 School of Biosciences, University of Nottingham, Sutton Bonington LE12 5RD, United Kingdom; 3 Neuroscience@Nottingham, University of Nottingham, Nottingham NG7 2RD, United Kingdom

**Keywords:** conditioning, fear, GABA, hippocampus, rat, salience

## Abstract

Hippocampal neural disinhibition, i.e., reduced GABAergic inhibition, is a key feature of schizophrenia pathophysiology. The hippocampus is an important part of the neural circuitry that controls fear conditioning and can also modulate prefrontal and striatal mechanisms, including dopamine signaling, which play a role in salience modulation. Consequently, hippocampal neural disinhibition may contribute to impairments in fear conditioning and salience modulation reported in schizophrenia. Therefore, we examined the effect of ventral hippocampus (VH) disinhibition in male rats on fear conditioning and salience modulation, as reflected by latent inhibition (LI), in a conditioned emotional response (CER) procedure. A flashing light was used as the conditioned stimulus (CS), and conditioned suppression was used to index conditioned fear. In experiment 1, VH disinhibition via infusion of the GABA-A receptor antagonist picrotoxin before CS pre-exposure and conditioning markedly reduced fear conditioning to both the CS and context; LI was evident in saline-infused controls but could not be detected in picrotoxin-infused rats because of the low level of fear conditioning to the CS. In experiment 2, VH picrotoxin infusions only before CS pre-exposure did not affect the acquisition of fear conditioning or LI. Together, these findings indicate that VH neural disinhibition disrupts contextual and elemental fear conditioning, without affecting the acquisition of LI. The disruption of fear conditioning resembles aversive conditioning deficits reported in schizophrenia and may reflect a disruption of neural processing both within the hippocampus and in projection sites of the hippocampus.

## Significance Statement

Hippocampal disinhibition, reduced GABAergic inhibition, is a feature of schizophrenia, but how this contributes to psychological deficits remains to be clarified. Patient studies using classical-conditioning assays show aberrant salience allocation to stimuli that healthy participants have learnt to ignore, as well as reduced fear conditioning, which have been linked to psychosis and negative symptoms, respectively. These impairments may be related to hippocampal disinhibition because the hippocampus modulates neural substrates of salience allocation and is part of the fear-conditioning neural circuit. Combining selective pharmacological manipulation of the hippocampus with a conditioning assay in rats, we found hippocampal disinhibition disrupted fear conditioning, without evidence for aberrant salience allocation. This suggests hippocampal disinhibition contributes to fear conditioning deficits in schizophrenia.

## Introduction

Hippocampal hyperactivity and neural disinhibition, i.e., reduced GABAergic inhibition, are key characteristics of schizophrenia pathophysiology and have been implicated in behavioral deficits characterizing the disorder (Friston et al., 1992; Lisman et al., 2008; Tamminga et al., 2010; Tregellas et al., 2014; Heckers and Konradi, 2015; Lieberman et al., 2018). This hyperactivity is most evident in the anterior hippocampus (McHugo et al., 2019), corresponding to the rodent ventral hippocampus (VH; Strange et al., 2014). Hippocampal disinhibition might contribute to behavioral impairments by disrupting neural processing both within the hippocampus, where regional disinhibition (by local microinfusion of the GABA-A receptor antagonist picrotoxin) causes aberrant burst firing (McGarrity et al., 2017) and alters oscillatory activity (Gwilt et al., 2020) in rats, and in hippocampal projection sites (Lodge and Grace, 2011; Bast et al., 2017; Katzel et al., 2020). Here, we tested whether hippocampal disinhibition contributes to deficits in latent inhibition (LI) and fear conditioning, which have been reported in schizophrenia.

LI refers to the reduced conditioning to a conditioned stimulus (CS), to which participants had been pre-exposed (PE) without consequence, and LI deficits have been reported in acute schizophrenia (Baruch et al., 1988; Gray et al., 1995; Rascle et al., 2001). One interpretation of reduced LI is that this reflects aberrant salience allocation to a stimulus that healthy participants had learned to ignore, and these findings contributed to the view that aberrant salience allocation is a key feature of schizophrenia and underlies psychotic symptoms (Gray et al., 1991; Kapur, 2003; Howes et al., 2020). Additionally, patients with schizophrenia show reduced aversive conditioning (Jensen et al., 2008; Holt et al., 2009; Romaniuk et al., 2010), which has been associated with negative symptoms (Holt et al., 2012).

The neural processes that underlie deficits in LI and aversive conditioning can be studied using rodent models. Permanent lesion studies in rats indicated that the hippocampus is not required for LI, although the adjacent entorhinal cortex and fibers passing through the hippocampus do play a role (Weiner, 2003); moreover, temporary inactivation studies indicated that the ventral subiculum may normally contribute to LI formation during pre-exposure (Peterschmitt et al., 2005, 2008). Interestingly, although NMDA-induced VH lesions spared LI acquisition, VH stimulation by local NMDA infusion moderately attenuated LI. However, this could partly have reflected reduced aversive conditioning (Pouzet et al., 2004). Although processing within the hippocampus could play a limited role in LI, VH stimulation and neural disinhibition might disrupt LI by stimulating dopamine release in ventral striatum and medial prefrontal cortex (mPFC; Legault et al., 2000; Mitchell et al., 2000; Floresco et al., 2001; Peleg-Raibstein et al., 2005; Bast, 2011). Increased dopamine function, especially in the ventral striatum (Joseph et al., 2000; Young et al., 2005; Nelson et al., 2011), but also the mPFC (Morrens et al., 2020), has been shown to disrupt LI at conditioning. Additionally, VH disinhibition disrupted mPFC-dependent attention, presumably by way of strong hippocampo-mPFC projections (McGarrity et al., 2017; Tan et al., 2018), and could also disrupt LI acquisition during CS pre-exposure, which has been shown to require the mPFC (Lingawi et al., 2018). Apart from LI, VH disinhibition may also disrupt aversive conditioning itself, because the VH contributes to fear conditioning (Bannerman et al., 2004; Fanselow and Dong, 2010) and VH stimulation by NMDA was found to disrupt fear conditioning (Zhang et al., 2001).

Therefore, we tested the hypothesis that VH disinhibition would disrupt the acquisition of LI and fear conditioning in rats. We determined the effect of VH neural disinhibition via local microinfusion of the GABA-A receptor antagonist picrotoxin (McGarrity et al., 2017) on LI and fear conditioning, using a conditioned emotional response (CER) procedure with a CS pre-exposure stage (Nelson et al., 2011). Experiment 1 examined VH disinhibition during both pre-exposure and conditioning; this markedly reduced fear conditioning to the CS, so we were unable to examine changes in LI. Therefore, experiment 2 examined the effect of hippocampal disinhibition during pre-exposure only on the formation of LI.

## Materials and Methods

### Rats

Overall, we used 104 male Lister Hooded rats (Charles River), weighing 310–400 g (9–12 weeks old) at the start of experiments. In experiment 1, 72 rats were tested in three batches of 24 rats. Experiment 2 used 32 rats in a single batch. See section below, Experimental design, for further detail and for sample size justifications.

Rats were housed in groups of four in individually ventilated “double decker” cages (462 × 403 × 404 mm; Techniplast) with temperature and humidity control (21 ± 1.5°C, 50 ± 8%) and an alternating 12/12 h light/dark cycle (lights on at 7 A.M.). Rats had *ad libitum* access to food (Teklad Global 18% protein diet, Harlan) throughout the study. Access to water was restricted during the CER procedure (see details below) but was available *ad libitum* during all other stages of the study. All rats were habituated to handling by experimenters for at least 5 d before any experimental procedure. All experimental procedures were conducted during the light phase and in accordance with the requirements of the United Kingdom Animals (Scientific Procedures) Act 1986, approved by the University of Nottingham’s Animal Welfare and Ethical Review Board (AWERB) and run under the authority of Home Office project license 30/3357.

### Stereotaxic implantation of guide cannulae into the VH

**Figure 1.**
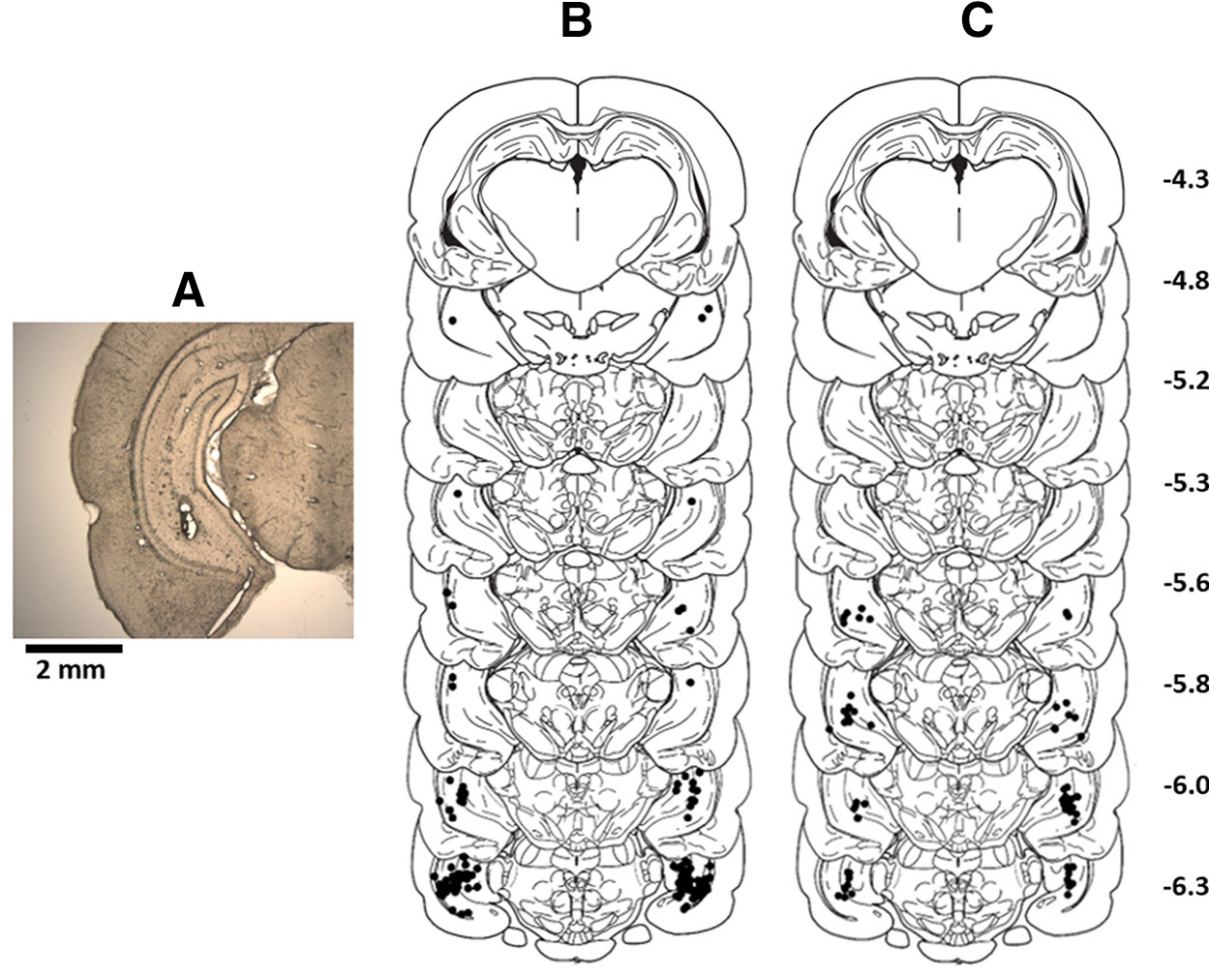
Infusion sites in the VH. ***A***, Illustrative coronal brain section showing infusion site in the VH. Approximate locations of infusion cannula tips (black dots) mapped onto coronal sections adapted from the Paxinos and Watson (1998) rat brain atlas for rats in experiments 1 (***B***) and 2 (***C***). Numbers on the right indicate posterior distance from bregma in millimeters.

Rats were anaesthetized using isoflurane delivered in oxygen (induced with 5% and maintained at 1.5–3%; flow rate 1L/min) and then placed in a stereotaxic frame. A local anesthetic (EMLA cream, AstraZeneca) was applied to the ear bars to minimize discomfort. A gel was used (Lubrithal; Dechra) to prevent the eyes from drying out during surgery. After incision of the scalp, bilateral infusion guide cannula (stainless steel, 26 gauge, 8.5 mm below pedestal, Plastics One) were implanted through small predrilled holes in the skull. The stereotaxic coordinates for the infusions were 5.2 mm posterior, ±4.8 mm lateral from the midline, and 6.5 mm ventral from the dura for infusions into the VH (see Fig. 1 for infusion cannula placements), based on previous studies targeting the VH in Lister Hooded rats (McGarrity et al., 2017). Stainless steel stylets (33 gauge, Plastics One), complete with dust cap, were placed into the guide cannula and protruded 0.5 mm beyond the tips of the guide cannula to prevent occlusion. Dental acrylic (flowable composite; Henry Schein Medical) and four stainless steel screws were used to fix the guide cannulae to the skull. The scalp incision was stitched around the acrylic pedestal to reduce the open wound to a minimum. All rats were injected with perioperative analgesia (Rimadyl, Large Animal Solution, Zoetis; 1:9 dilution; 0.1 ml/100 g, s.c.). At the end of surgery, rats were injected with 1 ml of saline (intraperitoneally) to prevent dehydration. Antibiotics were administered on the day of surgery and subsequently every 24 h for the duration of the study (Synulox; 140 mg amoxicillin, 35 mg clavulanic acid/ml; 0.02 ml/100 g, s.c.; Pfizer). After surgery, rats were allowed at least 5 d of recovery before any further experimental procedures were conducted. During this period, rats underwent daily health checks and were habituated to the manual restraint necessary for drug microinfusions.

### Microinfusions into the VH

Rats were manually restrained throughout the infusion process. Stylets were replaced with infusion injectors (stainless steel, 33 gauge, Plastics One), which extended 0.5 mm below the guide cannula tips into the VH. Injectors were connected via flexible polyethylene tubing to 5-μl SGE microsyringes mounted on a microinfusion pump (sp200IZ, World Precision Instruments). A volume of 0.5 μl/side of either 0.9% sterile saline (vehicle) or picrotoxin (150 ng/0.5 μl/side; Sigma-Aldrich) in saline was infused bilaterally over the course of 1 min, as in previous studies to induce neural disinhibition in the VH (McGarrity et al., 2017). The movement of an air bubble, which was included in the tubing, was monitored to ensure the solution had been successfully injected into the brain. Injectors were removed and replaced by the stylets 60 s after the end of infusion to allow for tissue absorption of the infusion bolus. The timing of infusions in relation to behavioral testing is described below, Experimental design.

In a previous study, the dose of picrotoxin (150 ng/0.5 μl/side) used did not cause seizure-related behavioral signs or electrophysiological signs of hippocampal seizures in local field potential recordings in anaesthetized rats (McGarrity et al., 2017). However, picrotoxin has the potential to cause epileptiform activity in the hippocampus (Qaddoumi et al., 2014). Therefore, all rats receiving infusions were monitored carefully during and after infusion for behavioral signs potentially related to seizure development, including facial twitching, wet-dog shakes, clonic limb movement, motor convulsions, and wild jumping (Racine, 1972; Luttjohann et al., 2009).

### CER procedure with a pre-exposure phase to measure aversive conditioning and its LI

**Figure 2.**
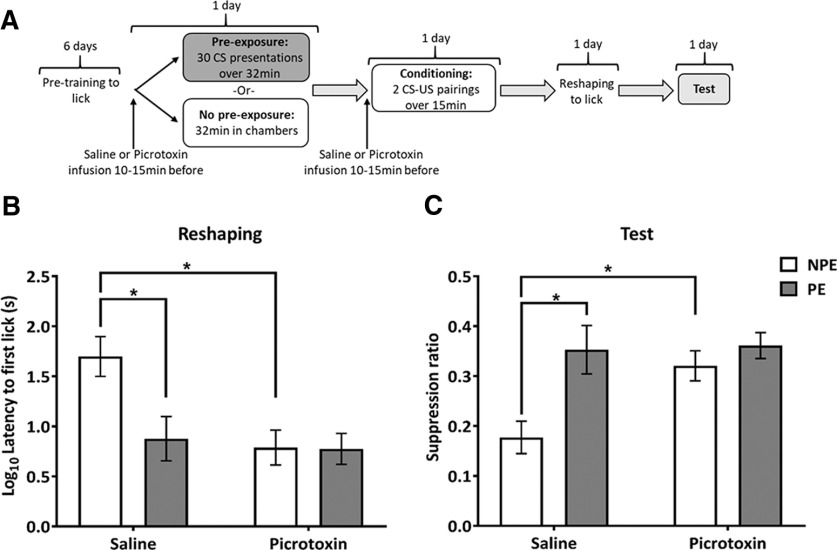
Experiment 1: ventral hippocampal disinhibition during pre-exposure and conditioning impairs the acquisition of contextual and elemental fear conditioning. ***A***, Design of experiment 1. ***B***, Mean (±SEM) latency to first lick values (s; log transformed) in the conditioning chamber following the aversive conditioning session for NPE (white bars) and PE (gray bars) rats in the saline and picrotoxin groups. Saline NPE rats show longer latencies compared with all other groups indicating increased conditioning to the conditioning context. Picrotoxin-infused rats show reduced latencies compared with saline-infused animals indicating impaired conditioning to the conditioning context. ***C***, Mean suppression ratio (±SEM) to the light CS for NPE (white) and PE (gray) rats in the saline and picrotoxin groups. Saline-infused rats displayed LI, with PE rats showing markedly less fear than NPE rats. Picrotoxin-infused rats show similarly low levels of fear conditioning in both NPE and PE groups reflecting picrotoxin infusion abolished conditioning to the CS. Asterisks indicate statistically significant differences between groups (*F *>* *9, *p* < 0.005; simple main effects analysis following significant interaction of infusion and pre-exposure).

**Figure 3.**
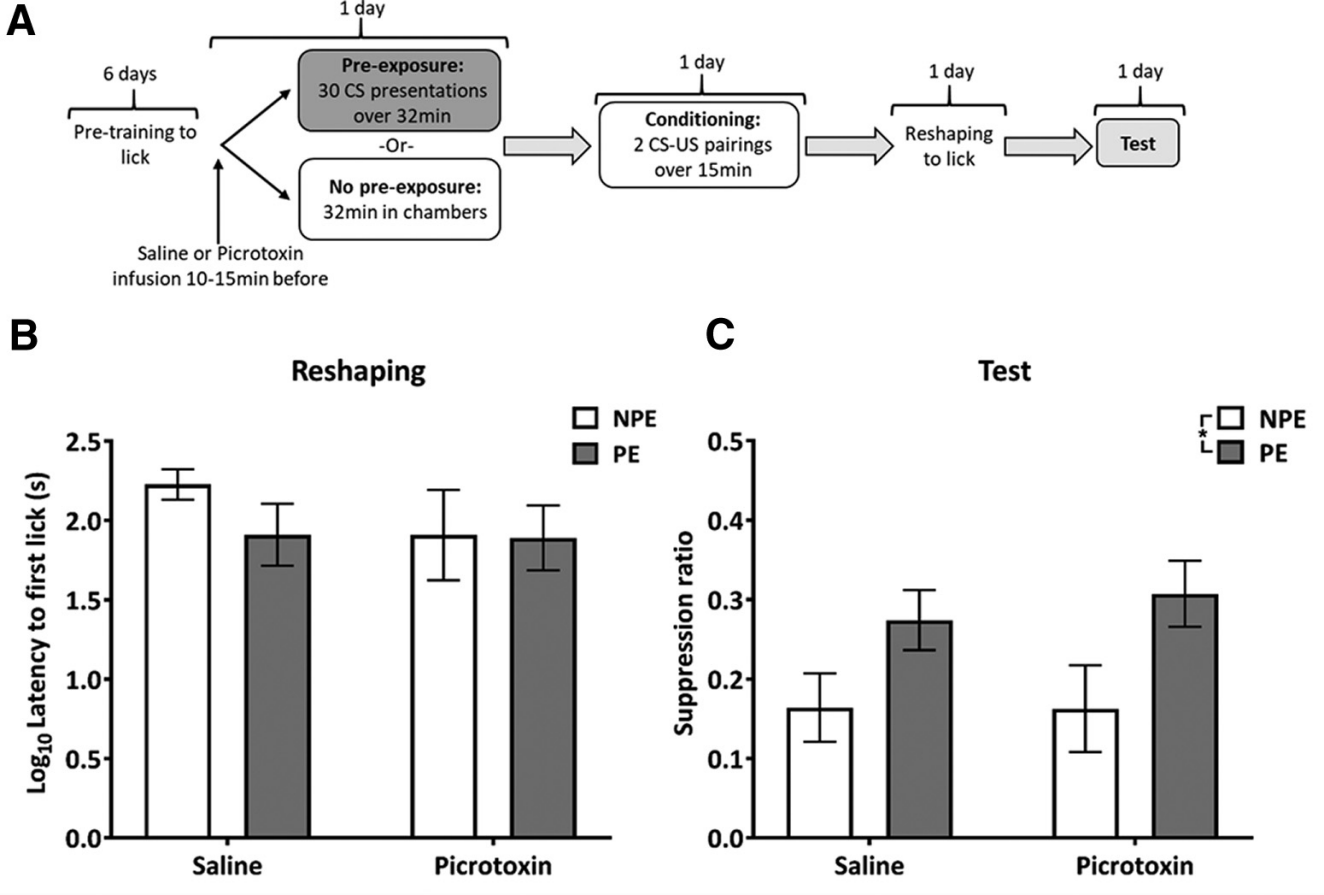
Experiment 2: VH disinhibition during pre-exposure does not impair the acquisition of LI. ***A***, Design of experiment 2, with the time point of the VH picrotoxin or saline infusion before the pre-exposure stage indicated. ***B***, Mean (±SEM) latency to first lick (s; log transformed) in the conditioning chamber, during reshaping, following the aversive conditioning session for NPE (white bars) and PE (gray bars) rats in the saline and picrotoxin groups. All groups show similar levels of contextual conditioning, indicated by similar latencies to first lick. ***C***, Mean suppression ratio (±SEM) to the light CS for control NPE (white) and PE (gray) rats in the saline and picrotoxin groups. Pre-exposure reduced fear responding to the CS in both saline and picrotoxin-infused rats compared with NPE rats, reflecting LI in both saline and picrotoxin-infused rats. Asterisk indicates significant main effect of pre-exposure during test (*F*_(1,24)_ = 8.44, *p *=* *0.008).

We used a CER procedure previously described by Nelson et al. (2011). The procedure, which will be described in detail below, involved water deprivation, shaping (1 d) and pretraining of the rats to drink from spouts in conditioning chambers (5 d), followed by pre-exposure to a light (the prospective CS) in conditioning chambers [or exposure to the conditioning chamber without CS pre-exposure in the non-PE (NPE) comparison group; 1 d], conditioning during which the CS was paired with an electric footshock, reshaping (1 d) to re-establish drinking after conditioning and testing (1 d) of the lick suppression induced by CS presentation following conditioning (for an outline of the CER stages, also see Figs. 2*A*, 3*A*). Suppression of licking for water by the CS was used to measure the CER. LI is reflected by a reduced CER, i.e., less suppression of licking for water, in the PE as compared with the NPE group.

#### Apparatus

Four identical fully automated conditioning chambers including sound attenuating cases and ventilation fans (Cambridge Cognition) were used. The inner chambers consisted of a plain steel box (25 × 25 × 22 cm) with a Plexiglas door (27 × 21 cm). The floor of the inner conditioning chamber comprised of a shock delivery system, consisting of 1-cm spaced steel bars. These were positioned 1 cm above the lip of a 7-cm-deep sawdust tray. Mounted 5 cm above the grid floor was a waterspout connected to a lickometer supplied by a water pump. Licks were registered by breaking a photo beam within the spout, which triggered water delivery of 0.05 ml per lick. The spout was only illuminated when water was available. Three wall mounted lights and the house light flashing on (0.5 s) and off (0.5 s) for 5 s functioned as the CS. Scrambled foot-shock of 1 mA intensity for 1 s provided the unconditioned stimulus (US). The shock was delivered through the grid floor by a constant current shock generator (pulsed voltage: output square wave 10 ms on, 80 ms off, 370-V peak under no load conditions; MISAC Systems). Stimulus control and data collection were recorded using an Acorn RISC computer programmed in basic with Arachnid extension (Cambridge Cognition).

#### Behavioral procedure

##### Water restriction

One day before behavioral testing, rats were water restricted for between 18 and 22 h. Subsequently, they received 1 h and 15 min of *ad libitum* access to water in their home cages for the duration of the experiment, once daily testing was completed and in addition to access to water in the conditioning chambers.

##### Shaping and pretraining

Rats were shaped for 1 d until all rats drank from the waterspout and were assigned an individual conditioning chamber for the whole CER procedure. Subsequently, rats were given a 15 min session (timed from first lick) per day for 5 d to drink from the waterspout. During the sessions, the waterspout was illuminated throughout, but no other stimuli were present. Total number of licks was recorded during each session to assess any pre-existing differences in drinking before infusions.

##### Preexposure

The PE rats received 30 5-s flashing light CS presentations with an average inter-stimulus interval of 60 s (32-min session duration). The NPE control rats were confined to the conditioning chamber for an identical period of time without receiving any CS presentations. Water was not available during the session and the waterspout was not illuminated.

##### Conditioning

One day after pre-exposure, rats were conditioned by two light-foot shock pairings, with the foot shock (1 mA/1 s) delivered immediately following the termination of the flashing light (5 s). The first light-shock pairing was presented after 5 min had elapsed and the second pairing 5 min after the first, followed by a further 5 min in the chamber, resulting in an overall session duration of 15 min. Water was not available during the session and the waterspout was not illuminated for the duration of the session.

##### Reshaping

The day after conditioning, rats were reshaped using the same procedure as used during the initial shaping. This was to re-establish drinking behavior after the conditioning session. Latency to first lick during reshaping was used as a measure of contextual fear conditioning to the chamber (Nelson et al., 2011, 2013).

##### Test

The day after reshaping, rats underwent a test session to assess conditioning to the CS. During the test session, water was available throughout, and the waterspout was illuminated. Once the rats had performed 50 licks, the CS was presented continuously for 15 min. The time taken to complete 50 licks before CS presentation (excluding latency to first lick) provides a measure of individual baseline variation (A period). This time was compared with the time taken to complete 50 licks during CS presentation (B period). A suppression ratio [A/(A + B)] was used to assess the overall level of conditioning to the CS, adjusted to individual variation in drinking, where a higher ratio represents a low level of fear conditioning (with a value of 0.5 or higher indicating no conditioning at all) and a ratio closer to 0 represents a high level of conditioning to the CS (Nelson et al., 2011, 2012).

### Verification of cannula placements

After behavioral experiments, rats were deeply anaesthetized with sodium pentobarbital (Dolethal, Vetoquinol) and were transcardially perfused with 0.9% saline followed by 4% paraformaldehyde in saline. Subsequently brains were removed and stored in 4% paraformaldehyde. Brains were sliced at 80-μm thickness using a vibratome and placed on microscope slides. Injector placements were identified using light microscopy and mapped onto coronal sections of a rat brain atlas (Paxinos and Watson, 1998).

### Experimental design

Both experiments 1 and 2 were run in a between-subjects design with a target sample size for both experiments of 16–18 per group. This sample size would give a power of >80% to detect effect sizes of Cohen’s *d *=* *1 for differences between groups (using between-subjects pairwise comparisons, two-tailed, with a significance threshold of *p *<* *0.05; G*Power; Faul et al., 2007), which has been suggested to be appropriate for neurobiological studies of aversive conditioning (Carneiro et al., 2018). Experiment 1 was run in three identical series, each including 24 rats. Experiment 2 was planned to comprise of two series, each containing 32 rats, but was ended after the first series. The second series was unnecessary, as there was clearly no evidence that the target effect size the study would have been powered for could be achieved (Neumann et al., 2017).

Rats were allocated to experimental groups according to a randomized block design. Two of the four rats in each cage were randomly assigned to the saline and the other two to the picrotoxin infusion group, and subsequently one rat of each pair was randomly assigned to either PE or NPE groups. The experimenters were blinded with respect to the infusion group allocation at the start of the experiment. In both experiments, several rats had to be excluded from the analysis of the whole experiment or some later stages of the experiment. During experiment 1, 13 rats fell ill, with presumed meningitis, before reshaping, while a further two rats fell ill after reshaping and before the test session; two additional rats had blocked guide cannulae after surgery and before behavioral testing, resulting in exclusion from the experiment; another rat showed extended convulsive seizures after picrotoxin infusion before conditioning. During experiment 2, one rat died during surgery and a further three rats fell ill, with presumed meningitis, before the reshaping session. The final sample sizes contributing to the analysis of performance measures at the different test stages in experiments 1 and 2 are shown in Table 1.

**Table 1:** Final number of rats included in data analysis per group for each stage of both experiments

	Experiment 1		Experiment 2	
Group	Reshaping	Test	Reshaping	Test
Saline NPE	14	14	7	7
Saline PE	13	10	8	8
Picrotoxin NPE	15	15	6	6
Picrotoxin PE	17	17	7	7

In experiment 1, VH drug infusions took place before both pre-exposure and conditioning sessions (Fig. 2*A*), whereas, in experiment 2, drug infusions took place before pre-exposure only (Fig. 3*A*). Rats were infused in batches of two pairs, by two experimenters, with each pair including one rat to receive saline and one rat to receive picrotoxin infusions. The two experimenters infused one pair, then the second pair, and testing started 10 min after the infusions for both rats of the second pair had been completed. This meant that all rats had a 10- to 15-min period between the end of the infusion and the start of behavioral testing. The timing of behavioral procedures after intracerebral infusions was based on electrophysiological measurements taken during VH infusion of picrotoxin (McGarrity et al., 2017) to capture the peak effect of hippocampal picrotoxin on neuronal firing following infusion.

### Statistical analysis

The measures taken during the CER experiments were analyzed using a 2 × 2 ANOVA with between-subject factors of pre-exposure group (NPE/PE) and drug infusion (saline/picrotoxin). All statistical tests and graphs were completed using SPSS (version 23), JASP (JASP Team: version 0.12.2, 2020) and GraphPad prism (version 7) software. The accepted level of significance was *p *<* *0.05. Raw latency data (time to first lick during reshaping) or time “A” data (time to 50 licks during test) were log transformed, as they showed unequal variance (Levene’s test, all *F *>* *5, *p *<* *0.002), to ensure a normal distribution and suitability for parametric analysis (Nelson et al., 2011, 2012).

## Results

### Cannula placements in the VH

In both experiments, all cannula tips were located within the VH, in coronal brain sections corresponding to between 4.3 and 6.3 mm posterior to bregma in the rat brain atlas by Paxinos and Watson (1998; Fig. 1). Many of the cannula placements, especially in experiment 1 (Fig. 1*B*) were located in the subiculum region of the VH, corresponding to the coronal section at 6.3 mm posterior to bregma in the atlas by Paxinos and Watson (1998) and similar to other studies targeting the VH (Bardgett and Henry, 1999; Bast et al., 2001b; McGarrity et al., 2017). We did not target a particular subregion of the VH, but note that the ventral subiculum together with the ventral CA1 region features overlapping functional connectivity to PFC and subcortical sites, including striatum, amygdala and septum (Groenewegen et al., 1987; Jay and Witter, 1991; Canteras and Swanson, 1992; Legault et al., 2000; Floresco et al., 2001; Dégenètais et al., 2003). As indicated in the Introduction and further considered in the Discussion, this functional connectivity is particularly relevant for the behavioral processes (fear conditioning and LI) investigated in the present study. It should also be noted that, although individual infusions may be placed in distinct subregions of the VH, such as subiculum or CA1, the drug will spread beyond one subregion within the VH. An infusion volume of 0.5 μl (as used in the present study) will occupy a sphere with a radius of 0.5 mm, if we assume isotropic spread of the infusion volume, and is likely to spread further, considering that spread is likely to be facilitated dorsally by the cannula tracks and diffusion will further add to drug spread (Jacobs et al., 2013). Previous multiunit electrophysiological recordings (McGarrity et al., 2017) showed that picrotoxin infusions into the VH, using the same coordinates as in the present study, resulted in marked enhancement of neural burst firing recorded by a multielectrode array straddling various subregions of the VH, including CA1, CA3, and dentate gyrus. In contrast, electrodes placed outside the medial and lateral boundaries of the VH did not reveal changes in neural firing, probably because of the densely packed fiber bundles surrounding the hippocampus (McGarrity et al., 2017). Therefore, the behavioral effects observed in the present study are likely to reflect disinhibition across several subregions of the VH, although disinhibition in subiculum and CA1 regions may be particularly important, given that these regions feature much of the relevant functional connectivity to prefrontal and subcortical sites. Because a substantial number of infusion sites in the present study were placed within the subiculum region of the VH, we include an additional analysis to explore whether key behavioral effects of VH disinhibition observed in the present study critically depended on cannula placements within the ventral subiculum.

### Experiment 1: VH disinhibition during preexposure and conditioning disrupts aversive conditioning

#### Pretraining

Analysis of latencies to lick at the end of pretraining, before pre-exposure, showed no overall effect of prospective infusion or pre-exposure group, nor an interaction of these factors (all *F*_(1,55)_ < 1; data not shown).

#### Reshaping

VH picrotoxin, compared with saline, infusion reduced latencies to first lick after reintroduction to the conditioning context during the reshaping session in the NPE group, which reflects reduced contextual fear conditioning. In the PE group both saline and picrotoxin groups showed similarly low levels of contextual conditioning, as measured by short latencies to lick, which indicates that pre-exposure to the light CS reduced contextual conditioning in the saline group (Fig. 2*B*). These observations were supported by a significant infusion × pre-exposure interaction (*F*_(1,55)_ = 4.7, *p *=* *0.034). Further examination of the interaction by simple main effects analysis showed that hippocampal picrotoxin, compared with saline, reduced conditioning in the NPE group (*F*_(1,55)_ = 11.9, *p *=* *0.001), but this was not apparent in the PE group, because of a floor effect where both saline and picrotoxin rats showed similarly low conditioning (*F*_(1,55)_ < 1). In addition, pre-exposure to the CS reduced context conditioning in saline-infused rats, reflected by reduced latencies in the PE group as compared with the NPE group (*F*_(1,55)_ = 9.0, *p *=* *0.004). This effect was not present in picrotoxin-infused rats (*F*_(1,55)_ < 1), probably reflecting a floor effect, i.e., the already low latencies in the picrotoxin rats.

#### Test

There was no difference in time to 50 licks before CS presentation (Time A) between infusion groups and pre-exposure groups (any effect or interaction involving infusion or pre-exposure: all *F *<* *3, *p *>* *0.09; data not shown). The group differences in latency to first lick that were evident at reshaping were not present during the test stage, probably reflecting extinguished contextual conditioning in the saline NPE group. The suppression ratios during the light test revealed that hippocampal disinhibition markedly disrupted conditioning to the CS in the NPE group, but did not affect conditioning in the PE group, i.e., there was no evidence that hippocampal disinhibition had affected LI (Fig. 2*C*). In saline-infused rats, the suppression ratio was markedly increased in the PE compared with the NPE group, reflecting reduced conditioning, i.e., LI (Fig. 2*C*, left). This difference between PE and NPE groups was not apparent in the picrotoxin-infused rats (Fig. 2*C*, right). However, this was because of picrotoxin-infused NPE rats showing markedly higher suppression ratios than saline-infused NPE rats, i.e., reduced conditioning to the light CS (compare white bars in Fig. 2*C*). In contrast, suppression ratios were similar in picrotoxin and saline-infused PE rats (Fig. 2*C*, compare gray bars). Thus, there was no evidence that hippocampal disinhibition reduced the impact of CS pre-exposure on conditioning. These observations were supported by a significant infusion × pre-exposure interaction (*F*_(1,52)_ = 4.142, *p *=* *0.047). Further examination of the interaction by simple main effects analysis revealed a main effect of infusion in the NPE group (*F*_(1,52)_ = 10.014, *p *=* *0.003) reflecting increased suppression ratio, i.e., reduced conditioning, caused by picrotoxin, compared with saline, whereas there was no effect of infusion in the PE group (*F*_(1,52)_ < 1). This resulted in the absence of a difference between PE and NPE in the picrotoxin-infused rats (*F*_(1,52)_ < 1), whereas saline-infused rats showed markedly higher suppression in the PE compared with the NPE group (*F*_(1,52)_ = 12.111, *p *=* *0.001).

### Experiment 2: VH disinhibition during preexposure alone does not affect conditioning or LI

#### Pretraining

Analysis of latencies to lick at the end of pretraining, before pre-exposure, showed no overall effect of prospective infusion (*F*_(1,24)_ = 2.9, *p *=* *0.104) or pre-exposure group (*F*_(1,24)_ < 1), and there was no interaction of these factors (*F*_(1,24)_ < 1; data not shown).

#### Reshaping

Hippocampal picrotoxin infusion only at pre-exposure had no effect on conditioning to the context, as reflected by latencies to first lick during reshaping, and there was no difference between pre-exposure groups (all main effects and interactions, *F*_(1,24)_ < 1.5, *p > *0.2; Fig. 3*B*). The latter contrasts with the finding in experiment 1, that pre-exposure reduced latencies to first lick in saline-infused rats (Fig. 2*B*).

#### Test

There were no differences in the A period (time to 50 licks before CS presentation) between infusion and pre-exposure groups (all main effects and interactions, *F*_(1,24)_ < 1.2, *p *>* *0.30; data not shown). Both drug infusion groups showed similar fear conditioning to the light CS, reflected by similar suppression ratios, and robust LI, reflected by higher suppression ratios in the PE compared with the NPE groups (Fig. 3*C*). This was supported by an effect of pre-exposure group (*F*_(1,24)_ = 8.44, *p *=* *0.0078), without a main effect or interaction involving infusion group (both *F*_(1,24)_ < 1).

### Seizure-related behavioral effects of hippocampal picrotoxin

In several rats receiving hippocampal picrotoxin infusions in experiment 1 (20 out of 32 rats receiving picrotoxin) and experiment 2 (six out of 15 rats receiving picrotoxin), we observed seizure-related behavioral signs, including facial twitching, wet-dog shakes and wild running, which can often be observed before full motor seizures (Racine, 1972; Luttjohann et al., 2009). These effects were observed within 5 min after the end of the picrotoxin infusion. They typically subsided within 30–45 min, after which rats showed no further adverse effects, with the exception of one rat, which showed continued uncontrollable clonic limb movement and was culled. We never observed these signs following saline infusions. Table 2 shows how many rats showed any of these seizure-related effects after the two picrotoxin infusions of experiment 1 or the one picrotoxin infusion of experiment 2. Although GABA network dysfunction, including in the hippocampus, is strongly implicated in the onset of seizures (Avoli and de Curtis, 2011), and the VH is a particularly seizure prone brain region, showing the earliest seizure activity in the pilocarpine rat model of seizures (Toyoda et al., 2013), previous studies using the same dose of picrotoxin as in the present study did not reveal seizure-related effects in Lister Hooded (McGarrity et al., 2017) or Wistar (Bast et al., 2001a) rats. Given that stress substantially facilitates hippocampal seizures (Joels, 2009; Manouze et al., 2019), the seizure-related effects of hippocampal picrotoxin infusions in the present study may reflect that, in contrast to previous studies involving hippocampal picrotoxin infusions, rats in the present study were exposed to water restriction and foot shocks as part of the CER procedure.

**Table 2:** Seizure-related behavioral signs observed after VH picrotoxin microinfusions

Observed behavior	Overall total	Experiment 1 total	Experiment 1		Experiment 2 total
			Infusion 1	Infusion 2	
Facial twitching	3	3	1	2	0
Wet dog shakes	19	15	11	7	4
Wild running	10	9	8	2	1
Clonic limb movement	1	0	0	0	1

The type of behavior observed is indicated in column one. Total number of rats experiencing seizure-related behavior signs overall during experiment 1 or 2 is shown in column two. The number of rats experiencing seizure-related signs during experiment 1 is detailed in column 3, with these signs separated to show the effects after the two individual infusions in columns 4 and 5. Column 6 details the total number of rats showing seizure-related signs after the one infusion of experiment 2.

**Figure 4.**
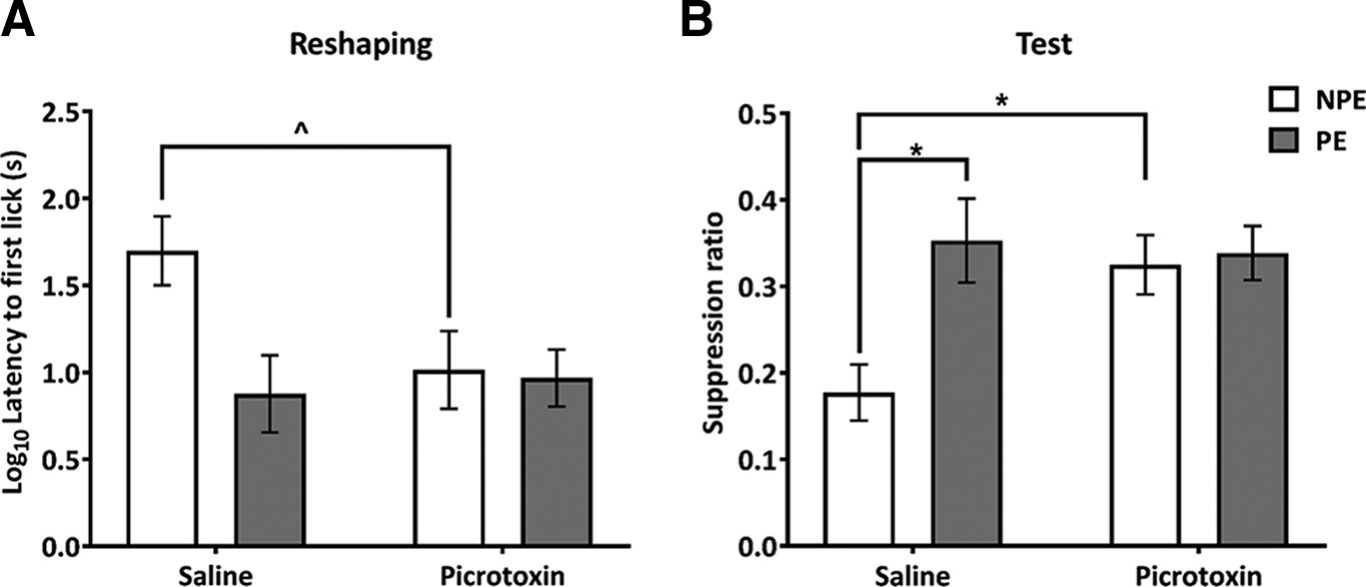
Experiment 1: an analysis limited to the rats that did not show seizure-related behavioral signs still reveals that ventral hippocampal disinhibition during pre-exposure and conditioning impairs the acquisition of contextual and elemental fear conditioning. ***A***, Mean (±SEM) latency to first lick values (s; log transformed) in the conditioning chamber following the aversive conditioning session for NPE (white bars) and PE (gray bars) rats in the saline and picrotoxin groups. Saline NPE rats show longer latencies compared with all other groups indicating increased conditioning to the conditioning context, similar to the pattern of results obtained from the whole sample (compare Fig. 2*B*). ***B***, Mean suppression ratios (±SEM) to the light CS for NPE (white) and PE (gray) rats in the saline and picrotoxin groups. Picrotoxin-infused rats show similarly low levels of fear conditioning in both NPE and PE groups reflecting picrotoxin infusion abolished conditioning to the CS, very similar to the pattern of results obtained from the whole sample (compare Fig. 2*C*); ^ indicates statistically significant differences between saline and picrotoxin infused NPE rats (*F*_(1,46)_* = *5.330, *p = *0.026; simple main effects analysis following a trend toward interaction of infusion and pre-exposure, *F*_(1,46)_ = 3.614, *p* = 0.0636). Asterisks indicate statistically significant differences between groups (*F* > 8, *p* < 0.01; simple main effects analysis following significant interaction of infusion and pre-exposure).

Importantly, additional analyses limited to the rats that did not show seizure-related behavioral signs during conditioning (saline NPE, *n* = 14; saline PE, *n* = 10; picrotoxin NPE, *n* = 10; picrotoxin PE, *n* = 13) still revealed a disruption of contextual and elemental fear conditioning in rats with VH disinhibition compared with saline-infused control rats in experiment 1 (Fig. 4). The pattern of changes in the measures of conditioning (latency to lick and suppression ratio, respectively) was virtually identical to the pattern revealed by the analysis including all rats (Fig. 2). More specifically, during reshaping, VH disinhibition in those rats that did not display seizure-related behavioral signs still reduced latencies to lick in the NPE group, reflecting reduced contextual fear conditioning (Fig. 4*A*). This was supported by a trend toward an interaction of infusion × pre-exposure (*F*_(1,46)_ = 3.614, *p *=* *0.0636) and a simple main effect of infusion in the NPE group (*F*_(1,46)_ = 5.330, *p *=* *0.026). In addition, during test, VH picrotoxin reduced conditioned suppression (i.e., increased the suppression ratio) in response to the light CS in picrotoxin-infused NPE rats as compared with saline-infused NPE rats in those rats that did not display seizure-related behavioral signs (Fig. 4*B*). This was supported by a significant interaction of infusion × pre-exposure (*F*_(1,43)_ = 4.933, *p *=* *0.0317) and a simple main effect of infusion in the NPE group (*F*_(1,43)_ = 8.310, *p *=* *0.006). Therefore, the disruption of fear conditioning by hippocampal disinhibition was not a consequence of seizure-related behavioral effects during conditioning.

### Placement of infusion sites in the ventral subiculum is not critical for the disruption of fear conditioning by VH disinhibition

**Figure 5.**
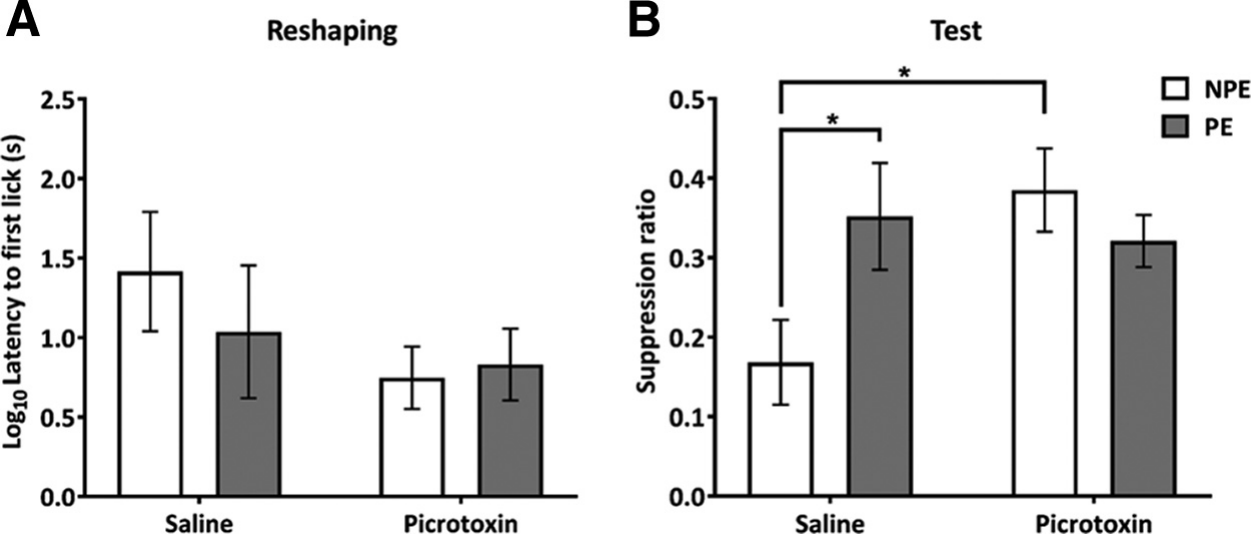
Experiment 1: data excluding rats with cannula placements in the ventral subiculum confirm that ventral hippocampal disinhibition during pre-exposure and conditioning impairs contextual and elemental fear conditioning. ***A***, Mean (±SEM) latency to first lick values (s; log transformed) in the conditioning chamber following the aversive conditioning session for NPE (white bars) and PE (gray bars) rats in the saline and picrotoxin groups. Picrotoxin-infused rats show numerically reduced latencies as compared with saline-infused rats, especially in the NPE groups, indicating impaired conditioning to the context, similar to the pattern of results from the whole sample (compare Fig. 2*B*). ***B***, Mean suppression ratio (±SEM) to the light CS for NPE (white) and PE (gray) rats in the saline and picrotoxin groups. Picrotoxin-infused rats show similarly low levels of fear conditioning in both NPE and PE groups reflecting picrotoxin infusion abolished conditioning to the CS. The pattern of results is very similar to the pattern obtained from the whole sample (compare Fig. 2*C*). Asterisks indicate statistically significant differences between groups (*F* > 6, *p* < 0.03; simple main effects analysis following significant interaction of infusion and pre-exposure).

To explore whether the marked impairments in fear conditioning caused by VH disinhibition in experiment 1 depended on whether the infusion sites were located in the subiculum or other subregions in the VH, we conducted an additional analysis, excluding data from rats with cannula placements in the ventral subiculum (i.e., placements within the coronal section corresponding to 6.3 mm posterior to bregma in Paxinos and Watson, 1998; see Fig. 1*B*). This analysis limited to rats with cannula placements outside the ventral subiculum (saline NPE, *n* = 5; saline PE, *n* = 5; picrotoxin NPE, *n* = 5; picrotoxin PE, *n* = 9) revealed that picrotoxin infusions still disrupted fear conditioning as compared with saline-infused rats (Fig. 5). The changes seen in this subset of rats were very similar to the changes in latency to lick and suppression ratio seen in the analysis that included all rats (Fig. 2). More specifically, during reshaping picrotoxin reduced latency to lick as compared with saline-infused rats, reflecting reduced contextual conditioning (Fig. 5*A*), although this difference did not reach statistical significance (main effect of infusion: *F*_(1, 22)_ = 2.076, *p *=* *0.1637) reflecting limited statistical power because of the reduced sample size. In addition, during test, picrotoxin infusions reduced conditioned suppression in response to the light CS in NPE rats as compared with saline-infused NPE rats (Fig. 5*B*). This was supported by a significant interaction infusion × pre-exposure: *F*_(1, 22)_ = 6.226, *p *=* *0.0206) and a simple main effect of infusion in the NPE group (*F*_(1,22)_ = 9.221, *p *=* *0.006). Overall, this analysis suggests that the fear conditioning deficits reported in experiment 1 were not exclusively mediated by picrotoxin infusions placed in the ventral subiculum, but rather that VH disinhibition by picrotoxin infusions placed in other subregions of the VH similarly caused fear conditioning deficits.

## Discussion

In experiment 1, VH disinhibition by picrotoxin during pre-exposure and conditioning markedly reduced fear conditioning to the CS and, therefore, any reduction of fear conditioning in the PE compared with NPE group, which would indicate LI, could not be detected. Picrotoxin and saline-infused rats in the PE group did not differ, showing similarly low conditioning, which does not support the hypothesis that hippocampal disinhibition affected salience modulation. In addition to disrupting conditioning to the CS, VH disinhibition also impaired contextual fear conditioning. In experiment 2, which specifically examined the impact of hippocampal disinhibition during pre-exposure alone, there was no evidence for any impact on LI.

### Preexposure-induced reduction of contextual fear conditioning

In experiment 1, the saline-infused PE rats showed shorter latencies to the first lick than NPE rats, reflecting reduced fear conditioning to the context. This could reflect that the novelty of the light stimulus enhanced memory formation (Lisman and Grace, 2005; King and Williams, 2009; Duszkiewicz et al., 2019) in the NPE group. The reduced context conditioning in PE compared with NPE saline-infused rats was not evident in experiment 2. This could be accounted for by a ceiling effect, i.e., higher levels of context conditioning, in experiment 2, which may have masked any further novelty-induced enhancement of context conditioning in the NPE group. In previous studies, un-operated rats showed stronger fear conditioning than cannulated rats that received hippocampal saline infusions, in terms of conditioned freezing (Zhang et al., 2001) and lick suppression (Zhang et al., 2000), suggesting that the infusion procedure itself, including the associated handling, might reduce fear conditioning. Therefore, the stronger conditioning in experiment 2 may partly reflect that, in contrast to experiment 1, the rats did not receive drug infusions immediately before conditioning.

### VH disinhibition during preexposure and conditioning markedly reduces fear conditioning without affecting LI

In experiment 1, VH disinhibition during both pre-exposure and conditioning markedly reduced fear conditioning to the CS in the NPE group, resulting in similarly low levels of conditioning in both the NPE and PE groups. Whilst there was no evidence for LI following VH disinhibition, the absence of LI was not because of increased conditioning in the PE group, which would reflect aberrant salience allocation, but instead was because of reduced conditioning in the NPE group. Similar to the present study, Pouzet et al. (2004), using a comparable LI paradigm, demonstrated VH NMDA stimulation reduced conditioned suppression in the NPE group, although there was also some evidence for disrupted LI with a trend toward greater conditioned suppression in PE compared with NPE rats. Moreover, studies in the prenatal methylazoxymethanol acetate (MAM) rat model of schizophrenia, which shows a loss of parvalbumin GABA interneurons and hyperactivity in the VH, also reported the absence of LI, which was mediated by reduced conditioning in the NPE group (Flagstad et al., 2005; Lodge et al., 2009).

### Disruption of elemental and contextual fear conditioning by VH disinhibition might reflect disruption of regional and distal processing

The impairments in fear conditioning to the CS and the context by VH disinhibition are likely mediated at the conditioning stage, which is supported by the finding in experiment 2 that disinhibition during pre-exposure alone did not affect conditioning. Impaired fear conditioning may reflect disrupted processing within the VH itself and in connected sites (Bast et al., 2017). Lesions, temporary inactivation by the sodium channel blocker TTX, and NMDA stimulation of the VH have been found to disrupt both contextual and elemental fear conditioning (Maren, 1999; Bast et al., 2001b; Zhang et al., 2001; Kjelstrup et al., 2002; Czerniawski et al., 2012). However, functional inhibition of the VH by the GABA agonist muscimol only disrupts contextual, but not elemental, conditioning, suggesting that neurons within the VH are mainly required for contextual fear conditioning (Bast et al., 2001b; Zhang et al., 2014). Therefore, the impaired contextual fear conditioning in the present study may reflect that disinhibition disrupts VH processing, whereas disrupted elemental fear conditioning is consistent with the idea that regional disinhibition can disrupt processing in VH projection sites (Bast et al., 2017), which have been implicated in elemental fear conditioning (see next paragraph). However, changes in dorsal hippocampal function, which is necessary for contextual fear conditioning and has been suggested to produce the underlying contextual representation (Anagnostaras et al., 2001; Bast et al., 2003; Matus-Amat et al., 2004; Hunsaker and Kesner, 2008), may also contribute to contextual fear conditioning deficits caused by VH disinhibition. VH disinhibition might disrupt dorsal hippocampal function by way of intrahippocampal inhibitory longitudinal connections (Sik et al., 1994, 1997). In line with this suggestion, a recent metabolic imaging study showed that VH disinhibition activated the VH but deactivated the dorsal hippocampus (Williams et al., 2019).

The VH also sends strong projections to the amygdala, mPFC, and septum (Risold and Swanson, 1997; Pitkanen et al., 2000; Cenquizca and Swanson, 2007; Hoover and Vertes, 2007), all of which are components of a brain circuit controlling conditioned fear responses to elemental stimuli (Tovote et al., 2015). The amygdala is a key component of the fear conditioning circuit and is thought to play a crucial role in the CS-US association and in conveying conditioned fear information to downstream effector sites (LeDoux, 2000; Duvarci and Pare, 2014). Thus, VH disinhibition, by causing aberrant drive of projections to the amygdala, could disrupt the processing of CS-US associations underlying conditioned fear. The mPFC is mainly thought to be required for the expression of cue conditioning and not its acquisition (Morgan et al., 1993; Pezze et al., 2003; Corcoran and Quirk, 2007), although inactivation of the rostral anterior cingulate cortex disrupted the acquisition of cue fear conditioning (Bissiere et al., 2008). The anterior cingulate cortex does not receive direct VH projections (Jay and Witter, 1991; Bian et al., 2019), but aberrant drive of VH projections to the mPFC might contribute to the disruption of elemental fear conditioning by way of regional connectivity within the mPFC (Jones et al., 2005). The lateral septum receives strong glutamatergic VH projections (Risold and Swanson, 1997; Cenquizca and Swanson, 2007) and is required for the acquisition of elemental fear conditioning (Calandreau et al., 2007). Additionally, hippocampo-lateral septum neurotransmission has been implicated in the modulation of the strength of CS-US associations and adaptive acquisition of conditioned fear responses (Desmedt et al., 2003; Calandreau et al., 2010). A recent neuroimaging study showed that VH disinhibition caused significant neural activation changes in the amygdala, mPFC, and LS (Williams et al., 2019) and, therefore, VH disinhibition could disrupt elemental fear conditioning by disrupting information processing at these projection sites.

In experiment 1, the VH was disinhibited during pre-exposure and conditioning, but not during reshaping and test. Therefore, the impaired fear conditioning evident during reshaping and test sessions could reflect state dependence, i.e., that information learned in one neural state can, in some cases, only be retrieved/expressed in the same state (Overton, 1964). To rule this out would require showing that fear expression is disrupted if the VH is disinhibited both during conditioning and the test expression of fear, but the interpretation of this finding would be difficult because the VH has been implicated in the expression of conditioned fear (Sierra-Mercado et al., 2011). However, several studies have shown that state dependent learning does not account for the conditioning deficits caused by local drug microinfusions into specific brain sites, including the mPFC, amygdala, and dorsal hippocampus (Guarraci et al., 2000; Bast et al., 2003; Pezze et al., 2003). In addition, previous experiments using a similar 3-stage fear conditioning paradigm to study systemic drug effects on LI found no evidence for state-dependent effects (Barad et al., 2004). Another possibility that deserves consideration is that the reduced conditioned fear during reshaping and test in experiment 1 could reflect that VH disinhibition disrupted reactivity to and processing of the electric footshock. However, previous studies reported that neither inactivation, via a sodium channel blocker or a GABA agonist (McEown and Treit, 2009, 2010), nor electrical stimulation (Dringenberg et al., 2008) of VH disrupted reactivity to electric footshocks. In addition, based on our own anecdotal observations, all rats across treatment groups similarly vocalized and flinched/jumped in response to foot shocks, although we did not systematically record and quantify these responses. Overall, a specific impairment in neural mechanisms underlying the formation of fear memory seems the most plausible account for the reduced conditioned suppression following VH disinhibition during conditioning.

### Hippocampal disinhibition during preexposure has no effect on the formation of LI

While aberrant dopamine transmission is thought to disrupt LI by interfering with the effect of pre-exposure during conditioning (Young et al., 2005; Morrens et al., 2020), stimulation and inhibition of GABA receptors disrupted LI formation at the pre-exposure stage (Feldon and Weiner, 1989; Lacroix et al., 2000). However, the lack of effect on LI acquisition by VH disinhibition during pre-exposure in experiment 2 suggests that sites outside the VH mediate the disruption of LI formation by systemic GABA receptor blockade during pre-exposure (Lacroix et al., 2000). Moreover, although VH disinhibition caused aberrant mPFC activation (Williams et al., 2019) and deficits in mPFC-dependent attention (McGarrity et al., 2017), our present findings show that VH disinhibition does not affect mPFC-dependent processing involved in LI formation during pre-exposure (Lingawi et al., 2017, 2018). In line with this, mPFC disinhibition during pre-exposure and conditioning did not disrupt LI formation (Enomoto et al., 2011; Piantadosi and Floresco, 2014). This is consistent with the idea that different prefrontal functions can display distinct relationships to prefrontal neural activity (Bast et al., 2017), with LI formation disrupted only by reductions (Lingawi et al., 2018), but not increases (Enomoto et al., 2011; Piantadosi and Floresco, 2014), in prefrontal activity, whereas sustained attention requires balanced levels of prefrontal activity (Pezze et al., 2014).

Although the present experiments do not support the hypothesis that VH disinhibition during pre-exposure affects LI, deactivation of the ventral subiculum during pre-exposure disrupted LI in a conditioned taste aversion paradigm, demonstrated by increased conditioning in the PE group (Peterschmitt et al., 2005, 2008). This suggests that LI formation normally requires the ventral subiculum during pre-exposure, but not balanced levels of ventral hippocampal activity.

### Clinical relevance

Our findings do not support the hypothesis that VH disinhibition disrupts LI and thus do not provide evidence to suggest that hippocampal GABA dysfunction contributes to LI impairments in schizophrenia. However, acute pharmacological disruption of GABA-A receptor mediated inhibition by picrotoxin as used in the present study does not fully capture all aspects of anterior hippocampal GABA dysfunction present in schizophrenia (e.g., chronicity, disruption in GABAergic interneuron function, rather than postsynaptic GABA receptor dysfunction, etc.) and, thus, further work is required to elucidate how some of these aspects of hippocampal GABA dysfunction may impact on salience modulation. Apart from impairments in LI and other aspects of salience modulation (Roiser et al., 2009, 2013), fear conditioning deficits have been reported in schizophrenia (Holt et al., 2009, 2012). Such deficits were suggested to contribute to difficulties in differentiating relevant from irrelevant stimuli (Hofer et al., 2001; Jensen et al., 2008) and were associated with negative symptoms (Holt et al., 2012). Previous findings have implicated prefrontal disinhibition in aversive conditioning deficits in schizophrenia (Piantadosi and Floresco, 2014). Our findings suggest that hippocampal disinhibition also contributes to deficits in aversive conditioning.

## Synthesis

Reviewing Editor: Jonathan Lee, University of Birmingham Decisions are customarily a result of the Reviewing Editor and the peer reviewers coming together and discussing their recommendations until a consensus is reached. When revisions are invited, a fact-based synthesis statement explaining their decision and outlining what is needed to prepare a revision will be listed below. The following reviewer(s) agreed to reveal their identity: David Bilkey.

The reviewers and reviewing editor agree that the paper presents findings that potentially advance the field. The importance of ventral hippocampal GABAergic inhibition to conditioning and latent inhibition is an interesting and relevant question. The experiments are well-designed and, for the most part, appropriately analysed. We particularly commend the authors on the clarity, detail and openness of the methods and results.

While the full reviews are appended below I would like to highlight the points that deserve particular attention.

1. While we again commend the openness of reporting of seizures and the exploratory analysis that excludes those animals observed to have suffered seizures, the discussion would warrant a more in-depth treatment of whether other animals might have had seizures that were not observed by the experimenters and whether sub-threshold abnormal activity might have occurred in the majority of animals and may therefore be an alternative explanation for the results. We would like a graphical representation of the data from the subset of animals not observed to have suffered seizures, as this may speak to the above question and would be of value to the reader.

2. Many infusion sites are at AP -6.3, and as such are consistent with published studies that treat these sites as being in the Ventral Subiculum. As the VH and VS may not serve common functions in conditioning and LI, this warrants at least some careful and detailed discussion. We also would suggest the merits of an exploratory analysis of injection site, distinguishing between the more anterior and posterior sites.

<i>Reviewer 1 comments</i>

This is a clearly written paper, that addresses an interesting question. The general methodology is appropriate and the results are analysed adequately. There are, however, aspects of the manuscript that lead me to question the findings.

1) First, a large number of the injection sites are located in the ventral subiculum (VS) at around AP -6.3, although the authors do not point this out. Although the subiculum is part of the hippocampal structure, it is fairly clear that the authors did not intend this as they treat this region differently to the hippocampus in their text (e.g. page 5). A comparison with the microinjection sites of Peterschmitt et al 2005 who injected into the VS suggest that they were very similar to those described in the current manuscript as ventral hippocampus in the current study. It is possible that the intention in the current paper was to inject further anterior, as a 5.5mm posterior site at the brain surface (as used) would have produced a genuine ventral hippocampal injection if the cannula had been inserted on the same plane as used in the Paxinos and Watson reference atlas.

2) Since there is some variance in the position of the microinjection it would be of interest to know if this was related to the effect (or lack of). Also some of the microinjection locations were very close to other structures so without this kind of analysis or some control we do not know whether any effect was due to hippocampal disinhibition or effects on other regions.

3) The above aside, a large proportion of the animals displayed visible symptoms of seizures. Once these occur there is the possibility of wide-ranging damage and disruption which could last for several days or longer. For this reason we cannot trust the data from these animals. The remaining animals might not have shown visible signs of seizures but without electrophysiology, there is the possibility that abnormal activity might have occurred.

4) It was concerning that a large number of animals developed meningitis (page 14). Was this due to a lack of infection control around the surgery?

<i>Reviewer 2 comments</i>

SUMMARY: The authors present two experiments addressing the contribution of vHPC to fear conditioning and LI. When GABAA receptors in the vHPC were blocked (using the compound picrotoxin) prior to preexposure and conditioning, contextual and cued fear expression were diminished. Because fear was so greatly diminished in the NPE group, the impact of this manipulation on LI specifically was unclear. A second experiment was conducted to test the hypothesis that GABAergic inhibition in the vHPC contributes to learned irrelevance by blocking these receptors prior to preexposure only. This manipulation had no effect on LI or fear expression. While these experiments are thoroughly described, I have a few concerns that I hope can be addressed prior to publication.

1. The authors do not discuss or rule out the possibility that picrotoxin may directly affect shock sensitivity or nociception. Do the authors have any data to speak to this (e.g., citing previous literature, scoring videos of unconditioned reactions to footshock, etc?) The main conclusions from the paper center around the discrepancy between the loss of fear in experiment 1 (with disinhibition overlapping with fear conditioning), and the maintenance of fear in experiment 2 (with disinhibition not overlapping with fear conditioning). Thus, the possibility that hippocampal control of “pain” is central to the interpretation.

2. Were rats habituated to the infusion procedure with “mock” infusions? This is a concern that may be related to the loss of the protective effect of PE on contextual fear in experiment 2, as compared to experiment 1, whereby rats in experiment 1 had more exposure to the infusion procedure and thus may have been more habituated, and less fearful. In terms of experimental design, it would have been prudent to “mock” infuse animals in experiment 2 prior to conditioning.

3. The authors use the term “disinhibition” to describe the effects of picrotoxin. While I believe this is warranted, the authors should explicitly discuss evidence that picrotoxin disinhibits cortical/allocortical activity / downstream neural activity to support their assertion.

4. The way the significance indicators are done in Fig 2C does not look like a main effect of pre-exposure group (the significance indicators look to me like the difference is comparing the between-subjects factor (Treatment), collapsed across the pre-exposure factor). This is likely because the authors want to keep the figure orientation the same as in Fig 1C, which makes sense. Perhaps a bracket and a star next to the legend for PE and NPE would be a better way of displaying the statistical significance?

5. The difference in the latency to first lick in Experiment 1 is couched in language related to an enhancement of memory in the NPE group. But, couldn’t the difference be driven by a loss of conditioning in the PE group due to learned irrelevance, which does not necessarily imply an “enhancement” of memory in the NPE group?

6. I commend authors on reporting specifics about rat illnesses / deaths, and for analyzing the seizure-like behaviors so thoroughly. I believe graphically displaying the conditioning and LI data from the subset of rats that did not display seizures would be beneficial.

7. The authors make a number of statements relevant to schizophrenia that I find problematic:

• In the abstract, the authors state that [“hippocampal neural disinhibition may contribute to impairments in fear conditioning [...] reported in schizophrenia", and then, “To test this hypothesis [...]” The authors are not testing the hypothesis that neural disinhibition contributes to impairments in fear conditioning in schizophrenia - they are testing whether GABAergic disinhibition affects fear conditioning and salience attribution only.

• Similarly, the “Clinical relevance” section of the discussion begins, “Our findings do not support the hypothesis that VH disinhibition contributes to LI impairments in schizophrenia”. There are many reasons that a single dose of a pharmacological compound delivered into a subregion of the hippocampus in a rat at specific time points prior to a behavioral task would fail to affect LI - none of them necessarily rule out the possibility that changes in GABAergic function in the anterior hippocampus of individuals with schizophrenia impact LI.

• The authors state “"The disruption of fear conditioning resembles aversive conditioning deficits reported in schizophrenia and may reflect disruption of neural processing within the hippocampus and its projection sites.”

Can the authors clarify the meaning of this statement? The effect in this study is by definition due to VH disruption - if the point is that this implies some deficit in this same circuit may be present in schizophrenia, that needs to be more clearly stated.

## Author Response

We are very pleased that the editor and reviewers found our manuscript to be of interest, and we thank them for their constructive comments. We have carefully considered the comments in order to improve the manuscript.

Importantly, as suggested by the two recommendations in the Synthesis of Reviews, we have revised the manuscript to include:

i) a graphical representation of the data from the subset of animals not observed to have suffered seizures, alongside a more detailed description of these data.

ii) an exploratory analysis of the impact of injection site, distinguishing between the more anterior and posterior sites.

Our responses to all comments by the two reviewers, alongside a detailed description of all changes made to the manuscript, are included below.

With our revision, we included a copy of the manuscript with all new text highlighted.

REVIEWER 1 COMMENTS

This is a clearly written paper, that addresses an interesting question. The general methodology is appropriate and the results are analysed adequately. There are, however, aspects of the manuscript that lead me to question the findings.

Author response: We thank the reviewer for these positive comments on our manuscript. We have carefully considered all points raised by the reviewer and thoroughly revised our manuscript in light of the comments.

1) First, a large number of the injection sites are located in the ventral subiculum (VS) at around AP -6.3, although the authors do not point this out. Although the subiculum is part of the hippocampal structure, it is fairly clear that the authors did not intend this as they treat this region differently to the hippocampus in their text (e.g. page 5). A comparison with the microinjection sites of Peterschmitt et al 2005 who injected into the VS suggest that they were very similar to those described in the current manuscript as ventral hippocampus in the current study. It is possible that the intention in the current paper was to inject further anterior, as a 5.5mm posterior site at the brain surface (as used) would have produced a genuine ventral hippocampal injection if the cannula had been inserted on the same plane as used in the Paxinos and Watson reference atlas.

Author response: We agree that many injection sites, especially in Exp. 1 (Fig. 1B), were placed in the subiculum region of the ventral hippocampus (VH) (corresponding to the coronal section at 6.3 mm posterior to bregma in the atlas by Paxinos and Watson, 1998), similar to other studies targeting the VH (Bardgett and Henry, 1999; Bast et al., 2001b; McGarrity et al., 2017). We did not target a particular subregion of the VH, but note that the ventral subiculum together with the ventral CA1 region features much of the same functional connectivity to prefrontal cortex and subcortical sites, including striatum, amygdala and septum. As indicated in the Introduction and further considered in the Discussion of our manuscript, this functional connectivity is particularly relevant for the behavioural processes (fear conditioning and latent inhibition (LI)) investigated in the present study. It should also be noted that, although individual injections may be placed in distinct subregions of the VH, such as subiculum or CA1, the drug will likely spread beyond one subregion within the VH. An injection volume of 0.5 μl (as used in the present study) will occupy a sphere with a radius of 0.5 mm, if we assume isotropic spread of the injection volume, and is likely to spread further, considering that spread is likely to be facilitated dorsally by the cannula tracks and diffusion will further add to drug spread (Jacobs et al., 2013). Previous multi-unit electrophysiological recordings (McGarrity et al., 2017) showed that picrotoxin injections into the VH, using the same coordinates as in the present study, resulted in marked enhancement of neural burst firing recorded by a multi-electrode array straddling various subregions of the VH, including CA1, CA3 and dentate gyrus. In contrast, electrodes placed outside the medial and lateral boundaries of the VH did not reveal changes in neural firing, probably because of the densely packed fibre bundles surrounding the hippocampus (McGarrity et al, 2017). Therefore, the behavioural effects observed in the present study are likely to reflect disinhibition across several subregions of the VH, although disinhibition in subiculum and CA1 regions may be particularly important, given that these regions feature overlapping functional connectivity to prefrontal and subcortical sites. To clarify these issues, we have now added to our description and discussion of the infusion sites in our revised manuscript (p. 14-16, ‘Cannula placements in the VH’).

2) Since there is some variance in the position of the microinjection it would be of interest to know if this was related to the effect (or lack of). Also some of the microinjection locations were very close to other structures so without this kind of analysis or some control we do not know whether any effect was due to hippocampal disinhibition or effects on other regions.

Author response: We agree with the reviewer and recommendation 2 in the review synthesis that the spread of injection sites offers the opportunity to explore if the marked impairments in fear conditioning caused by VH disinhibition in Exp. 1 depended on whether the injection sites were located in the subiculum or other subregions in the VH. To this end, we conducted an additional analysis, excluding data from rats with cannula placements in the ventral subiculum (i.e., placements within the coronal section corresponding to 6.3 mm posterior to bregma in Paxinos and Watson, 1989; see Fig. 1B). When we removed animals whose infusion tip locations encroached on the ventral subiculum, the pattern of results is the same as revealed by analysis of the whole data set: picrotoxin infusions reduced contextual conditioning (as reflected by reduced latency to lick at reshaping in the picrotoxin compared to the saline rats) and cue conditioning (as reflected by a greater suppression ratio in NPE rats infused with picrotoxin compared to NPE rats infused with saline). This additional analysis is reported in a new section of our revised Results section (p. 21/22, Placement of injection sites in the ventral subiculum is not critical for the disruption of fear conditioning by VH disinhibition), and the data are presented in our new Figure 5.

For additional considerations concerning the spatial selectivity of the effects of disinhibition targeting the VH, please see our response to comment 1).

3) The above aside, a large proportion of the animals displayed visible symptoms of seizures. Once these occur there is the possibility of wide-ranging damage and disruption which could last for several days or longer. For this reason we cannot trust the data from these animals. The remaining animals might not have shown visible signs of seizures but without electrophysiology, there is the possibility that abnormal activity might have occurred.

Author response: In our original manuscript, we had already included a statistical analysis limited to the rats that did not show seizure-related behavioural changes. In the revised manuscript, we have also included a graphical representation (new Figure 4) of the data for those rats that did not display seizure-related behavioural signs and expanded on our description of these results (p. 20/21) (as requested by reviewer 2, comment 6, and in recommendation 2 of the review synthesis). The pattern of changes in the measures of conditioning (latency to lick and suppression ratio) revealed by this analysis was virtually identical to the pattern revealed by the analysis including all rats, supporting that seizure-related effects do not account for the impairments in fear conditioning.

Previous electrophysiological studies do not support that picrotoxin infusions that do not induce seizure-related behavioural signs cause seizure-related changes in hippocampal neural activity. More specifically, as stated on p. 8 (first paragraph) of the manuscript, previous electrophysiological studies under anaesthesia did not reveal epileptic-like brain activity within the VH (McGarrity et al 2017). Instead, as described on p. 3, first paragraph, picrotoxin infusion into the VH caused a marked enhancement of neural burst firing and more subtle alteration of hippocampal LFP oscillations within the VH (McGarrity et al., 2017; Gwilt et al., 2020).

4) It was concerning that a large number of animals developed meningitis (page 14). Was this due to a lack of infection control around the surgery?

Author response: All surgical procedures were performed using full aseptic techniques, in line with UK Home Office guidance and as approved by our Named Veterinary Surgeon. The rats developed illness several weeks after surgery. This suggests that illness was not due to infection during the surgical procedures, but was likely due to infection caused over time through the trepanation of the skull. For subsequent studies, we altered our surgical approach to make use of light-cured dental cement, as opposed to solvent based curing, in order to achieve better ‘sealing’ around the trepanation. This significantly reduced the number of rats that fell ill.

REVIEWER 2 COMMENTS

SUMMARY: The authors present two experiments addressing the contribution of vHPC to fear conditioning and LI. When GABAA receptors in the vHPC were blocked (using the compound picrotoxin) prior to preexposure and conditioning, contextual and cued fear expression were diminished. Because fear was so greatly diminished in the NPE group, the impact of this manipulation on LI specifically was unclear. A second experiment was conducted to test the hypothesis that GABAergic inhibition in the vHPC contributes to learned irrelevance by blocking these receptors prior to preexposure only. This manipulation had no effect on LI or fear expression. While these experiments are thoroughly described, I have a few concerns that I hope can be addressed prior to publication.

Author response: We thank the reviewer for the positive and constructive comments on our manuscript. We have carefully considered the points raised for the revision of our manuscript.

1. The authors do not discuss or rule out the possibility that picrotoxin may directly affect shock sensitivity or nociception. Do the authors have any data to speak to this (e.g., citing previous literature, scoring videos of unconditioned reactions to footshock, etc?) The main conclusions from the paper center around the discrepancy between the loss of fear in experiment 1 (with disinhibition overlapping with fear conditioning), and the maintenance of fear in experiment 2 (with disinhibition not overlapping with fear conditioning). Thus, the possibility that hippocampal control of “pain” is central to the interpretation.

Author’s response: We agree with the reviewer that this is an important issue, which would benefit from some discussion, and have now added discussion of this issue to the manuscript (p. 27). Previous studies reported that neither inactivation, via a sodium channel blocker or GABA agonist (McEown and Treit, 2009, McEown and Treit, 2010), nor electrical stimulation (Dringenberg et al., 2008) of VH or dorsal hippocampus disrupted reactivity to footshocks. In addition, based on our own anecdotal observations, all rats across treatment groups similarly vocalised and flinched/jumped in response to foot shocks, although we did not systematically record and quantify these responses.

2. Were rats habituated to the infusion procedure with “mock” infusions? This is a concern that may be related to the loss of the protective effect of PE on contextual fear in experiment 2, as compared to experiment 1, whereby rats in experiment 1 had more exposure to the infusion procedure and thus may have been more habituated, and less fearful. In terms of experimental design, it would have been prudent to “mock” infuse animals in experiment 2 prior to conditioning.

Author’s response: The rats were not habituated to the infusion procedure prior to conditioning in Experiment 2. We agree that, in hindsight, this approach may indeed have been preferential, as mock infusions would have meant rats in Experiment 2 would have experienced similar infusion-related handling before fear conditioning as the rats in Experiment 1. This issue is discussed in the section on ‘Pre-exposure-induced reduction of contextual fear conditioning’ section (Page. 23), where we explain that overall reduced conditioning in Experiment 1, as compared to Experiment 2, may be due to the infusion procedure, including associated handling of the rats.

3. The authors use the term “disinhibition” to describe the effects of picrotoxin. While I believe this is warranted, the authors should explicitly discuss evidence that picrotoxin disinhibits cortical/allocortical activity / downstream neural activity to support their assertion.

Author’s response: In the Abstract (first line) and at the beginning of our Introduction (first line), we define ‘neural disinhibition’ as ‘reduced GABAergic inhibition’. We also consider the effect of hippocampal picrotoxin infusion in rats on neural activity within the hippocampus in the first paragraph of the introduction, stating that in the hippocampus ‘regional disinhibition causes aberrant burst firing (McGarrity et al., 2017) and alters oscillatory activity (Gwilt et al., 2020) in rats’ (p. 3). We appreciate that, from this statement it was not clear that this referred to neural disinhibition by local picrotoxin infusion. Therefore, we have clarified this in the revised manuscript. In addition, as stated in our Discussion, a whole-brain metabolic imaging study demonstrated that VH picrotoxin infusions activated the VH (p. 25, first paragraph, last sentence) and changed neural activity in projection sites of the VH (p. 26, first paragraph, last sentence).

4. The way the significance indicators are done in Fig 2C does not look like a main effect of pre-exposure group (the significance indicators look to me like the difference is comparing the between-subjects factor (Treatment), collapsed across the pre-exposure factor). This is likely because the authors want to keep the figure orientation the same as in Fig 1C, which makes sense. Perhaps a bracket and a star next to the legend for PE and NPE would be a better way of displaying the statistical significance?

Author’s response: We think the reviewer refers to Fig. 3C (not 2C). We agree that altering the figure and legend would be a better way to represent the significant main effect of pre-exposure and have now changed this to clarify the meaning of the significance indicator (Fig. 3C).

5. The difference in the latency to first lick in Experiment 1 is couched in language related to an enhancement of memory in the NPE group. But, couldn’t the difference be driven by a loss of conditioning in the PE group due to learned irrelevance, which does not necessarily imply an “enhancement” of memory in the NPE group?

Author’s response: We do not quite understand this comment. Learned irrelevance refers to reduced conditioning to a conditioned stimulus (CS) as a result of previous uncorrelated exposure of the CS and unconditioned stimulus (US). However, in our paradigm, there never is an uncorrelated exposure of relevant CSs (light or context) and the US (instead rats are only pre-exposed to light and context, in the pre-exposed, PE, group, or only to the context, in the non-preexposed, NPE, group). So, we do not understand how the development of learned irrelevance may contribute to less contextual fear conditioning in the PE group. If the reviewer wishes us to address this comment, could they please clarify how they think learned irrelevance may contribute to less context conditioning in the PE group?

6. I commend authors on reporting specifics about rat illnesses / deaths, and for analyzing the seizure-like behaviors so thoroughly. I believe graphically displaying the conditioning and LI data from the subset of rats that did not display seizures would be beneficial.

Author’s response: Following the reviewer’s suggestion and recommendation 1 in the review synthesis, we have now included a graphical representation of the subset of rats that did not show seizure-related behavioural signs (new Figure 4). We have also expanded on our original description of this data in the revised Results section of our manuscript (p. 20/21).

7. The authors make a number of statements relevant to schizophrenia that I find problematic:

• In the abstract, the authors state that [“hippocampal neural disinhibition may contribute to impairments in fear conditioning [...] reported in schizophrenia", and then, “To test this hypothesis [...]” The authors are not testing the hypothesis that neural disinhibition contributes to impairments in fear conditioning in schizophrenia - they are testing whether GABAergic disinhibition affects fear conditioning and salience attribution only.

Author’s response: We agree that the accuracy of these statements can be improved. In the revised manuscript, we have attempted to amend this (Abstract, lines 5 to 7).

• Similarly, the “Clinical relevance” section of the discussion begins, “Our findings do not support the hypothesis that VH disinhibition contributes to LI impairments in schizophrenia”. There are many reasons that a single dose of a pharmacological compound delivered into a subregion of the hippocampus in a rat at specific time points prior to a behavioral task would fail to affect LI - none of them necessarily rule out the possibility that changes in GABAergic function in the anterior hippocampus of individuals with schizophrenia impact LI.

Author’s response: We have now revised this statement to improve its accuracy (p. 29).

• The authors state “"The disruption of fear conditioning resembles aversive conditioning deficits reported in schizophrenia and may reflect disruption of neural processing within the hippocampus and its projection sites.”

Can the authors clarify the meaning of this statement? The effect in this study is by definition due to VH disruption - if the point is that this implies some deficit in this same circuit may be present in schizophrenia that needs to be more clearly stated.

Author’s response: The point we wish to highlight is that the fear conditioning impairment caused by VH disinhibition may reflect not only disruption of processing within the VH, but also of processing within projection sites of the VH. We have rephrased our statement in the Abstract in an attempt to clarify this (Abstract, last sentence).
